# Neck and Musculoskeletal Pain Among Dentists: A Review of the Literature

**DOI:** 10.7759/cureus.33609

**Published:** 2023-01-10

**Authors:** Abed AlRaouf Kawtharani, Ammar Chemeisani, Fadi Salman, Ali Haj Younes, Ali Msheik

**Affiliations:** 1 Internal Medicine, Lebanese University Faculty of Medicine, Hadath, LBN; 2 Neurological Surgery, Lebanese University Faculty of Medicine, Hadath, LBN; 3 General Surgery, Lebanese University Faculty of Medicine, Hadath, LBN; 4 Obstetrics and Gynaecology, Lebanese University Faculty of Medicine, Hadath, LBN; 5 Neurological Surgery, Faculty of Medical Sciences, Lebanese University, Hadath, LBN

**Keywords:** periodontics, neck pain index, postural pain, neck pain, dentistry

## Abstract

Musculoskeletal disorders (MSD), notably neck pain, are important occupational health issues in the field of dentistry. Many studies were done worldwide to gather data about neck and back pain. They used different characteristics and risk factors. Other studies aimed to determine only the prevalence of neck and back problems among dentists. We aim to review the literature for research about the optimal factors to be assessed and the proper measures to be installed by dentists to prevent cervical pain and to be taught and shared with dental students. Such an aim requires a thorough review of the current condition. This is a brief review of the literature to shed light on the latest news on this topic. Research using keywords such as dentistry, neck pain, neck pain index, occupational pain, and dental specialties were used to skim the literature for related topics. Publications are considered based on their relevance to the topic. Topics related to other professions and pain induced by illnesses other than occupational factors are not included. The final conclusion shows that no final results were conducted regarding the optimal characteristics that the dentists should be using. Therefore, we recommend a study with an aim to determine the optimal factors to be used by dentists to prevent cervical pain and to be taught to dental students.

## Introduction and background

According to the World Health Organization, around 1.71 billion people have musculoskeletal conditions around the world. Musculoskeletal disorders (MSD), notably neck pain, are important occupational health issues in the field of dentistry [1]. These problems go way back in time and are still highly spread. In a study conducted among dentists in 1990, 72% had pain and discomfort from either the neck, shoulders, or headaches [2]. In another study in 2018 among dental professionals in Western countries, 58.5% had neck pain, 56.4% had lower back pain, 43.1% had shoulder pain, and 41.1% had upper back pain [3]. Musculoskeletal disorders can be defined as “a group of diseases and complaints that affect different structures of the musculoskeletal system, including the nerves, tendons, muscles, joints, ligaments, bones, blood vessels, and supporting structures such as intervertebral discs” [3].

There is no sure evidence of how the pain is distributed between women and men; females reported more neck pain than men according to studies conducted in Germany while no significant differences were determined in a study in Iran [4,5]. Musculoskeletal disorders are, however, more commonly observed among younger dentists than older practitioners in both men and women [2]. More importantly, it is crucial to grasp the meaning of musculoskeletal pain in order to widen understanding of its causes, risk factors, and prevention methods, improve diagnosis and management, and raise awareness of the ergonomic factors related to dentists’ musculoskeletal health and overall well-being. Having a healthy musculoskeletal system is indeed crucial to the dentistry profession, as it is physically and mentally demanding [3].

This review aims to review the impact of neck pain on the ability and productivity of dentists’ practice. Prevalence, incidence, morbidity, and long-term findings ought to be reviewed through the literature to shed light on the current data available. It is mandatory that we consider the data gap present in the literature for recommendations for future studies. Furthermore, it aimed to assess the disability level in dentists and evaluate the benefits of preventive actions in decreasing pain severity through the review of the literature for surveys and assessment studies if any are valid and available.

## Review

Dentist work and general health

A dentist’s job is an interaction between two parties, helper and recipient [6]. Dentists aid patients by diagnosing their dental issues and treating them, then advising them on how to develop better oral hygiene regimens. Responsibilities fall upon dentists if they want to practice dentistry, which can be summarized as diagnosing oral diseases and infections, promoting general oral health, restoring patients’ oral health through adequate treatment methods, monitoring the development of teeth and jaw, and performing oral surgical procedures [7]. There are several dental specialties and training [7]. Endodontics is the specialty of diagnosing, preventing, and treating diseases and injuries of the dental pulp and surrounding tissues. Orthodontics and dentofacial orthopedics is the specialty of diagnosing, intercepting, and correcting dental and facial irregularities. Pediatric dentistry is the specialty of diagnosing and treating the oral health care needs of infants and children through adolescence. Periodontics is the specialty of diagnosing and treating diseases of gum tissue and bones supporting teeth. Finally, prosthodontics is the specialty of restoring natural teeth or replacing missing oral structures with artificial devices such as dentures.

Dentists are subject to several physical and psychological disorders throughout their time of practice [6]. Physical disorders primarily include musculoskeletal problems, dermatoses, allergies, and possible cross-infection. Psychological disorders contribute greatly to affecting the dentist’s health, and factors include job-related stress, tension, depression, emotional exhaustion, and depersonalization [6].

Musculoskeletal pain

Before diving into the definition of musculoskeletal pain and disorders, it is important to understand that the musculoskeletal system is constituted of bones, muscles, ligaments, tendons, cartilage, nerves, and blood vessels [8]. In simple terms, musculoskeletal disorders are injuries or disorders of the musculoskeletal system [9]. These include a range of inflammatory and degenerative conditions but are not limited to sprains, inflammation, strains, bone splintering, and stress fractures. These disorders are associated with pain and usually progress over time [10]. Musculoskeletal disorders occur mostly during occupational activities and influence the efficacy and the health of workers in general and dentists specifically, in addition to bringing on economic burdens on the country because of compensation for work-related injuries [11].

As for neck pain, studies have defined neck musculoskeletal disorders in two ways, one which considers symptoms occurring in the neck, and the other considering symptoms in addition to physical examination findings. Ergonomic exposure observations and measurements should be made specific to the neck region such as neck posture, neck angle measurements, neck work-load assessment, and others [12].

In order to understand the factors affecting the muscles, an understanding of the anatomy and physiology of head and neck musculature is necessary. In the back arises the cervical portion of the vertebral column, which is a structure referred to as a column of spools, curved in a ventral direction. Then, we have the globe-like head, which teeters on two half-round occipital condyles, and the head lies in front of them. With the presence of the muscles in the back of the neck (attached to an occipital bone), the head is held erect and is restrained from collapsing. In the front, some major muscles contribute to the tensions, which are the masticatory, suprahyoid, and infrahyoid muscle groups, which are linked in chains, joining the cranium with the mandible, the mandible with the hyoid bone and the latter with the shoulder girdle [13]. In addition, it is informative to note that musculoskeletal disorders are distinguished from other conditions, such as cancer, as symptoms appear while on the job between what is estimated to be six to 12 months after starting working and contribute to the formation of chronic pain [14].

Musculoskeletal disorders (MSDs)

Following the previous definition, it is notable that some disorders can be asymptomatic or transitory, and others can be very severe and may lead to disability. Acute and chronic conditions can emerge from these disorders, the chronic ones being more frequent with a prevalence of 30% to 40% [8]. Musculoskeletal disorders are characterized as being associated with repeated trauma and can be classified into clinically well-defined disorders such as tendinitis and hand-arm vibration, less-clinically defined disorders such as tension and neck syndrome, and non-specific such as repetitive strain injuries and cervicobrachial disorders. Other musculoskeletal disorders include degenerative disk disease, thoracic outlet compression, epicondylitis, and mechanical back syndrome. Musculoskeletal conditions are a high contributor to the global need for rehabilitation. In 2017, the World Health Organization launched the Rehabilitation 2030 initiative in order to raise awareness on the matter of musculoskeletal disorders and create a strategic approach to rehabilitation from these disorders. Indeed, most countries were not qualified to respond to rehabilitation needs, so this initiative came into use for suffering patients. In some low- and middle-income countries, more than half of people with musculoskeletal disorders do not receive the rehabilitation they need [15].

Health effects

According to the World Health Organization, musculoskeletal disorders are a leading cause of disability worldwide, as they have a significant impact on limiting mobility and dexterity [15]. Musculoskeletal disorders range in effect from causing intermittent pain that doesn’t necessarily affect job performance or efficacy, to severe conditions where the pain is prevalent most of the time and affects daily tasks [16]. The longer the exposition to work hazards, the more tissue deterioration progresses in the dentist. The repetitiveness of exposure is one of the main factors that affect the type of injury and symptom severity. Also, symptoms differ from one condition to the other, as some dentists will exhibit well-defined symptoms while others are less easily discernable (Table 1). Some common symptoms of musculoskeletal disorders and injuries are local or generalized pain, hypersensitivity, loss of muscle strength, inflammation, loss of balance, etc. These symptoms, in addition to other ones, can increase the severity of the condition over time and cause physical and psychological alterations in the performance and efficacy of the dentist in both his daily life tasks and work tasks. Unnatural posture, awkward movements, constant pain, muscle weakness, and reduction of cognitive capacities are a few of the effects of musculoskeletal disorders [16].

**Table 1:** Common MSDs; general signs and symptoms Adapted from Nermin Y et al. [8] MSD: musculoskeletal disorder

Disease manifesting as musculoskeletal pain	Symptoms of musculoskeletal pain	Reported physical findings
Inflammation of the sciatic nerve	Joint pain	Loss of muscle mass
Inflammation of the tendons	Joint edema	
Bilateral palm cyanosis	Peripheral numbness	Weakness of muscular power
Spinal disc herniation	Joint decreased range of motion	
Essential tremor	Hyperalgesia	Abnormal sensation
Inflammation of the epicondylar cartilage		
Tendon compression		

Dentists exhibit common movements, such as forward and lateral flexions, which compromise the tissues around the neck and thus create imbalances and reduce neuromuscular efficiency over time. When maintaining one posture or movement for a prolonged period of time, there is uneven engagement of the joints, and this impacts the motor engram of the dentists’ bodies, which usually aids them in adapting to forces and pressure. Talking about the neck specifically, its deep cervical flexors, the primary stabilizers of muscles, are impaired, which leads to the overworking of the superficial muscles such as the trapezius, thus increasing the symptoms of disorders in the neck and adding several triggers to it [17,18].

Risk factors

Risk factors are elements that may increase the risk of injury or disorders among practitioners during their occupational work. There are always clinically significant determinants of disease in every clinical disorder and thus, there are several factors that influence musculoskeletal disorders. In the following, we will be examining the different risk factors of musculoskeletal pain [8,17,19]. According to the American Dental Association, the risk factors of MSD can be summarized as repetitions, force, mechanical stresses, posture, vibration, cold temperature, extrinsic stress, and predisposing factors [19]. Repetitions can be defined as “the average number of movements or exertions performed by a joint or a body link within a unit of time” [19]. Over-extension and overuse of muscles is a side effect of repetitive movements, as it leads to overall fatigue of these muscles. Also, it is found that the antagonistic tendons and muscle groups are the ones that are touched and affected by symptoms. When one exerts a series of movements to accomplish a task, force is considered the physical effort in this process. The dentist’s work relies on movements of the hand, which is very often elevated and therefore causes more muscle fatigue. Mechanical stresses are defined as “impingement or injury by hard, sharp objects, equipment or instruments when grasping, balancing or manipulating” [19].

When dentists work with their wrists or forearms against the edge of a surface, the muscles and tendons are impinged, which creates mechanical stresses. For every joint in the body, there is a neutral zone of movement in which movements do not require high muscular force. If the dentist tried to accomplish a movement outside this zone and with an awkward or deviated posture, the risk of injury increases, and the presence of symptoms also increases. Bad posture includes strained sitting positions, bending forward, tipping shoulders, head tilts, and others. These are all related to the risk of developing musculoskeletal symptoms. Vibration as a risk factor has a small chance of increasing the presence of musculoskeletal symptoms. It is true that vibrations of equipment should ideally be between 20 to 80 Hz, however, they are on average from 5000 to 10000 Hz. Since they are used for relatively short periods of time, they do not have a huge impact on the musculoskeletal system. Mechanical vibrations seem to affect the body through the upper limbs by causing changes in the vascular, neural, and osteoarticular systems. This can lead to a disease called vibration syndrome [19,20]. According to Silverstein in his lecture on work-related musculoskeletal disorders, low temperatures in general can reduce dexterity and increase nerve-end impairment in the body [19]. Extrinsic stresses include job variety, job control, workload, time pressure, and financial constraint. Predisposing Factors include biological mechanisms (age, hormonal imbalances, etc.), and other numerical factors such as weight or wrist size.

In sum, some harmful factors play a role in the emergence of neck and musculoskeletal pain in the organism. These causes are considered multifactorial, which means that they depend on several factors. The biomechanics of seated working postures, the unidirectional twisting of the trunk, working in a monotone position for long periods of time, the dentist’s flexibility and core strength, and the dentists knowing how to adjust ergonomic equipment have a relationship with the emergence of physiological damage and pain, including neck pain.

In a study conducted in Kuwait in 2021 to study the risk factors for work-related musculoskeletal disorders for dentists, the results showed that dentists suffering from musculoskeletal symptoms worked longer hours than those who did not experience pain, and this is thought to be due to prolonged and awkward postures that subject the dentists to unnatural forces and stresses on crucial movement and functioning joints [21]. Sartorio et al. found that awkward postures, intense work schedules, prolonged repetitive movements, and the dentist’s workstation are all risk factors for MSD. In addition, it is believed that factors related to professional equipment, such as vibrations, also affect MSD in dentists [22].

Prevention and treatment

In a study in 2015 aiming to assess the efficacy of an exercise program for neck pain relief in dentists practicing in Tehran, results showed that eight weeks of exercise therapy decreased neck pain among dentists significantly. It is recommended that dentists frequently change their body positions, decrease the static activity of muscles, increase the frequency of breaks between sessions, and fix posture with the aim of lessening the risk of MSDs. The mentioned study showed that dentists who usually were physically active complained less about their neck pain than those who did not exercise. However, these exercises did not target specific muscles, and thus these dentists were not getting the ultimate benefits of exercising. The authors mentioned several benefits of exercising, such as strengthening muscles, increasing blood flow, oxygen, and nutrient supply to muscular cells, and in turn, preventing the risk of MSDs. In addition, pain-inhibiting hormones are secreted during exercise, which helps relieve muscle pain among exercising dentists [23].

A systematic review of nine databases aimed to clarify the physiological effects and benefits of stretches used to reduce musculoskeletal disorders. Results showed that stretching reduces discomfort and pain and is most beneficial when combined with other measures to remediate the causes of pain; otherwise, it may result in the suppression of awareness of the injury. Also, one should be careful when stretching, as it can aggravate an injury if inadequately performed [24].

The principles of ergonomics

According to the World Health Organization, one should create “an appropriate balance between the requirements of the work and the capacity of the working person.” The dentist should then consider his working capacity and adapt it to the working conditions of his profession. Also, he should follow ergonomics principles for the dentistry profession in order to decrease the risk of musculoskeletal disorders and pain such as fixing posture, etc. [25]. The Centers for Diseases Control and Prevention (CDC) states that employees should participate in ergonomic training in order to be familiar with ways to prevent illness. In addition, they should use ergonomic assistive devices, such as products (slip sheets, slings, mechanical or electronic hoists), and equipment (adjustable beds, grab bars) [26].

Interventions were also made by the International Standards Organization (ISO #6385) defining the fundamental principles of ergonomics as basic guidelines for the design of work systems. These should be used in the case of MSDs. They are workspaces and equipment that should be adapted to the nature of work and preferred body postures should be performed during work, sufficient space should be provided for body movements, machinery should be implemented in a way to assist or execute repetitive work, and extreme posture should be avoided when exerting high force movements [27].

Pain and productivity of dentists

Muralidharan, Fareed, and Shanthi (2013) conducted a study to determine the prevalence and distribution of musculoskeletal disorders among dental practitioners in a city in the southern state of Andhra Pradesh in India. Out of 73 dental practitioners, 78% of participants had a prevalence of at least one MSD symptom over the past 12 months, with the most affected areas being the neck (52%), the lower back (41%), shoulders (29%), and wrist (26%). MSD prevalence was highest among orthodontists (100%) and oral physicians (100%). Sick leaves were required for 40% of the practitioners in the last 12 months. MSDS resulted in doctor consultations, sick leaves, hospitalizations, alteration of duties, as well as a reduction in work activity and leisure activity. The study concluded that a high prevalence of MSDs exists among dental practitioners, which affects the daily practice of more than one-third of them [28].​​​​

M*usculoskeletal Pain Among Dentists*

A study conducted in Greece in 2014 investigated the prevalence and characteristics of musculoskeletal disorders among Greek endodontists in the past 12 months, in addition to the treatment followed by their postures during practice and the adoption of ergonomic standards. Among 147 dentists, 61% reported MSDs with a prevalence of lower back pain (30%) and neck pain (30%). Awkward postures during practice, regular stretching exercises, and the number of patients per day were significant predictors of MSDs. Only 53% had sought medical care for their disorder(s) [29].

In another study in Queensland Australia conducted in 2004 on 400 dentists, of which 73.3% were male and 26.7% were female, 87.2% of dentists reported at least one musculoskeletal symptom in the past year, and neck pain was the most prevalent (57.5%) [1]. This study also showed that subjects with back pain reported more neck pain and hand/wrist pain than those without back pain. Also, age and gender were seen to be significant for neck pain, and senior people and women suffered from neck pain more than others. Almost one in 10 dentists reported taking leave in the previous 12 months because of an MSD. Among those who took sick leave for an MSD, the mean time taken was 11.5 days [1].

A study conducted in Riyadh among dental professionals in Riyadh working in governmental and private sectors studied the severity and extent of back and neck pain and assessed the related factors. Of the respondents, 53.7% reported lower back pain and 51.9% reported neck pain. Shoulder pain was also common (42.5%). Among those dentists, 44.2% exercised and 36.1% did not. In sum, age group, educational level, working sector, and exercise were associated with musculoskeletal pain. This study, unlike others, showed that musculoskeletal pain is independent of sex [30].

In Vojvodina in 2017, a study aimed at determining the presence of discomforts in areas of the head, neck, shoulders, upper back, and upper limbs at health professionals in the area of dentistry, as well as discomfort localization and methods of treatment. Seventy-five point nine percent (75.9%) of dentists, 90.9% of dental assistants, and 40% of dental technicians experienced pain around the neck area in addition to headaches in the occipital part [10]. Fifty-nine point four percent (59.4%) of the cases did not seek medical help.

A study conducted at Kerman Medical University in Iran in 2018 among dental students in the third, fourth, fifth, and sixth years comprised 199 students. It aimed to investigate ergonomic factors that cause muscle pain. Results of this research showed that over 69% of the students suffered from pain in at least one body area, with most suffering from hand and elbow (23%), and others from headaches (19%). Endodontics (79%) and pedodontics (73%) students were the most in pain. Also, males had higher levels of pain. Demographic data (sex, age, weight, height, sports, and smoking) were associated with the Rapid Entire Body Assessment score. This study also showed that only 20% of students had comfortable dental chairs. This study concluded that the students’ sitting positions and working environments needed to be improved with more ergonomic training [31].

Musculoskeletal Pain Among Dentists in the Lebanese Community

In Lebanon, dentists also suffer from musculoskeletal disorders and pain. A few studies aimed to evaluate this pain among dentists in the Lebanese community. In the first study of 314 Lebanese dentists, 61.5% had spinal pain, 31.6% had cervical pain, 22.3% had lumbar pain, and 13% had dorsal pain. It was shown to be continuous in 20.7% of dentists and occasional in 65.8% of them. In addition, 30.6% had frequent headaches. The researchers concluded from the results of this study that the occurrence of musculoskeletal pain in the upper extremities is a serious concern affecting dentists in Lebanon [32].

A second study aimed to assess the prevalence and relationships between demographic variables and some dental practices with musculoskeletal disorders among Lebanese dentists in 2016. It considered 218 dentists. Results showed that 92.7% of these dentists suffered from MSDs, and the most affected areas were the lower back (61.8%), neck (51.5%), shoulders (39.5%), fingers (14.1%), wrists (11.8%), and elbows (8.6%). There were also statistical differences between different dentistry specialties, for example, neck pain was highest among endodontic dentists while back pain was highest among pediatric dentists. Also, female dentists reported suffering more than male dentists concerning neck and wrist levels [33].

A third study evaluated the risk of developing musculoskeletal disorders in preclinical and clinical dental students in Beirut Arab University (BAU) clinics among 190 students. The methods used were the Ergonomic Awareness Questionnaire (EAQ) followed by the Rapid Entire Body Assessment Scale (REBA) scale. The results of these tests showed that 90% of preclinical and 90% of clinical dentistry students were at medium risk of developing musculoskeletal problems during practice, and the other 10% were at very high risk. In addition, 78% of preclinical students had a good awareness of ergonomics compared to 83% of clinical students, but only 44% of preclinical students and 62% of clinical students actually implemented ergonomic principles in their practice. Also, 58% of preclinical students and 81% of clinical students were found to suffer from musculoskeletal disorders [34].

Neck Disability Index

In 2005, Sterling et al. implemented revisions to the Neck Disability Index (NDI), which was first introduced in 1996 (Table 2) [35]. Later, Diaz-Caballero et al. implemented a validated questionnaire that was utilized widely in the literature for an assessment of the situation of neck pain among dentists and the ergonomics of this situation (Table 3) [36]. NDI is designed to understand how neck pain affects the ability to manage everyday life activities. The NDI is a 10-item scale following a Likert scale from 0 to 5 [37]. Of note, implementation of the NDI is limited in the literature and only two studies consider the utilization of the NDI for the assessment of neck pain in dentists [38,39].

**Table 2:** Questionnaire sections and questions per section with the corresponding choices

Questionnaire section	Question per section	Choices
Demographic Data	Gender	Male
		Female
	Age	25-35 years
		36-45 years
		46-56 years
		>56 years
	Type of practice	Governmental
		Private
		Both
	Specialty	General Dentist
		Periodontist Endodontist
		Prosthodontist
		Pedodontist
		Maxillofacial surgeon
		Orthodontist
	Hours of work per week	15-30
		31-40
		41-50
	Regular exercise	Yes
		No
	Experiencing pain using vibrating instruments	Yes
		No
	Cervical flexion for better vision while working	Yes
		No
	Do you have any history of those mentioned below? If yes, please select which one(s)	Congenital Spinal Disease
		Spine trauma
		Spine surgery
		None
Ergonomics	Do you have muscular pain due to dental practice?	Yes
		No
	Are you familiar with the ergonomic posture to perform clinical procedures in your dental practice?	Yes
		No
	Which activities of your clinical practice produce muscular pain? Mark the main activities	Surgery
		Endodontics periodontics
		Restorative
	Reference the previous question: Mark in which zone you feel the pain	Lumbar zone (lower back)
		Dorsal zone (mid-back)
		Cervical zone (upper back)
		Neck
		Shoulders
		Forearm
		Arm
		Wrist
		Hand
		Other:
	Are you able to change your work posture, seating or standing, during your practice?	Yes
		No
	Do you frequently change positions during your clinical practice?	Yes
		No
	After finishing clinical practice, do you perform stretching exercises?	Yes
		No
	Are the instruments within hand reach without making strenuous movements?	Yes
		No
	Do you perform torsions or cervical flexions to improve vision when working in the oral cavity?	Yes
		No
	Do you cross your legs while working?	Yes
		No
	On a scale of 0 to 10, what is the intensity of the neck pain?	No pain
		0
		1
		2
		3
		4
		5
		6
		7
		8
		9
		10
		Worst pain possible

**Table 3:** NDI and Likert scale conversion NDI: Neck Disability Index

N#	Item	Response	Score
NDI1	Pain intensity	I have no neck pain at the moment	0
		The pain is very mild at the moment	1
		The pain is moderate at the moment	2
		The pain is fairly severe at the moment	3
		The pain is very severe at the moment	4
		The pain is the worst imaginable at the moment	5
NDI2	Personal Care	I can look after myself normally without causing extra neck pain.	0
		I can look after myself normally, but it causes extra neck pain.	1
		It is painful to look after myself, and I am slow and careful.	2
		I need some help but manage most of my personal care.	3
		I need help every day in most aspects of self-care.	4
		I do not get dressed. | wash with difficulty and stay in bed.	5
NDI3	Lifting	I can lift heavy weights without causing extra neck pain.	0
		I can lift heavy weights, but it gives me extra neck pain.	1
		Neck pain prevents me from lifting heavy weights off the floor but I can manage if items are conveniently positioned, i.e. on a table.	2
		Neck pain prevents me from lifting heavy weights, but I can manage light weights if they are conveniently positioned.	3
		I can lift only very light weights	4
		I cannot lift or carry anything at all.	5
NDI4	Reading	I can read as much as I want with no neck pain.	0
		I can read as much as I want with slight neck pain.	1
		I can read as much as I want with moderate neck pain.	2
		I can’t read as much as I want because of moderate neck pain.	3
		I can’t read as much as | want because of severe neck pain.	4
		I can’t read at all.	5
NDI5	Headaches	I have no headaches at all.	0
		I have slight headaches that come infrequently.	1
		I have a fair degree of difficulty concentrating.	2
		I have a lot of difficulty concentrating.	3
		I have a great deal of difficulty concentrating.	4
		I can’t concentrate at all.	5
NDI7	Work	I can do as much work as I want.	0
		I can only do my usual work, but no more.	1
		I can do most of my usual work, but no more.	2
		I can't do my usual work.	3
		I can hardly do any work at all.	4
		I can't do any work at all.	5
NDI8	Driving	I can drive as long as | want without neck pain.	0
		I can drive as long as | want with only slight neck pain.	1
		I can drive as long as | want with moderate neck pain.	2
		I can't drive as long as | want because of moderate neck pain.	3
		I can hardly drive at all because of severe neck pain.	4
		I can't drive my car at all because of neck pain.	5
NDI9	Sleeping	I have no trouble sleeping.	0
		My sleep is slightly disturbed for less than 1 hour.	1
		My sleep is mildly disturbed for up to 1-2 hours.	2
		My sleep is moderately disturbed for up to 2-3 hours.	3
		My sleep is greatly disturbed for up to 3-5 hours.	4
		My sleep is completely disturbed for up to 5-7 hours.	5
NDI10	Recreation	I am able to engage in all my recreational activities with no neck pain at all.	0
		I am able to engage in all my recreational activities with some neck pain.	1
		I am able to engage in most, but not all of my recreational activities because of the pain in my neck.	2
		I am able to engage in a few of my recreational activities because of the pain in my neck.	3
		I can hardly do recreational activities due to neck pain.	4
		I can't do any recreational activities due to neck pain	5

With the implementation and adoption of the questionnaire introduced by Diaz-Carabello et al., several studies aimed to study the main pain zones in dentists. Aghahi, Darabi, and Hashemipour’s study showed that dental chairs, sitting positions, and working environments were factors associated with the prevalence of musculoskeletal pain and disorders [32]. Also, another study conducted by Ajwa et al. showed that age group, educational level, and exercise were also risk factors for musculoskeletal disorders [31]. In a study by Shaik et al., the researchers found there was a significant association between working hours per day and stiffness in the hand (P = 0.018) [37].

A cross-sectional study conducted among 184 dentists in Saudi Arabia reported a prevalence of MSD pain up to 90.2% and identified the following predictors for MSD pain: older age (OR 1.23; 95% CI 1.00 to 1.50) and female gender (OR 2.52; 95% CI 1.12 to 5.68), the time the dentist spent with patients (OR 0.28; 95% CI 0.14 to 0.54), and years of experience (p < 0.05) [40].

From these findings, it is clear that musculoskeletal pain is a burden on dentists, as they use a series of members and psychomotor skills to work and sit in static postures using precision hand and wrist movements, which, in the presence of MSDs, can turn out to be very challenging and painful.

## Conclusions

This review of the literature aimed to shed light on the current situation of neck pain among dentists worldwide and among Lebanese dentists. In addition, this review aimed to reveal the factors implemented in the pain and the recommendation to lessen the impact on the suffering dentists. It was evident that the time of working, the repetition of ergonomic movements, awkward postures, and the mechanical vibration installed by machinery used in dental procedures during long schedules are the main precipitators of neck pain. This review showed that neck pain reduces drastically the productivity of dentists in up to 40% of the cases. Studies elaborating on the condition of neck pain and Lebanese dentists provide the basis concerning the current situation and are advocative of studies to reveal the suffering index in this population of dentists.

Many studies were done worldwide to gather data about neck and back pain. They used different characteristics and risk factors such as the method of looking with or without a mirror and the type of treatment. Other studies aimed to determine only the prevalence of neck and back problems among dentists. No final results were conducted regarding the optimal characteristics that the dentists should be using. Therefore, it is advocated to determine the optimal factors to be used by dentists to prevent cervical pain and to be taught and shared with dental students. This must be done through a cross-sectional study with the implementation of the NDI. Future studies should consider conducting large-scale surveys to better investigate the risk factors associated with the presence of musculoskeletal pain in dentists, especially given that different studies seem to give inconsistent results. Also, some factors, other than ergonomic and socio-demographic characteristics, such as psychological factors, should be considered, which could have an impact on the development of physical pain and disease. Finally, a study by observation should be considered, if possible, to reduce the bias caused by self-reporting.
